# CX3CL1 in Early Detection of Alzheimer's Disease: Plasma Dynamics Across Age and Disease Stages

**DOI:** 10.1002/acn3.70320

**Published:** 2026-01-16

**Authors:** Ling Wang, Yujie Liu, Fei Li, Xuelin Li, Lanlan Li, Jie Zhang, Yali Xu

**Affiliations:** ^1^ Chongqing Medical University Chongqing China; ^2^ Department of Geriatrics, Chongqing General Hospital Chongqing University Chongqing China; ^3^ Chongqing Clinical Research Centre for Geriatric Diseases Chongqing China; ^4^ Department of Health Management, Chongqing General Hospital Chongqing University Chongqing China

**Keywords:** aging, Alzheimer's disease, amnestic mild cognitive impairment, cognitively normal, CX3CL1, inflammatory chemokines, plasma

## Abstract

**Backgrounds:**

Alzheimer's disease (AD) is characterized by amyloid‐beta plaques, tau tangles, and neuroinflammation. C‐X3‐C motif chemokine ligand 1 (CX3CL1, also known as fractalkine), a neuroimmune chemokine implicated in AD pathogenesis, shows inconsistent alterations in plasma/serum across studies. Specifically examining age‐dependency and diagnostic utility, we investigated plasma CX3CL1 levels across the cognitive continuum (cognitively normal [CN], amnestic mild cognitive impairment [aMCI], AD) in a Chinese cohort.

**Methods:**

A total of 443 participants, including 130 patients with AD, 72 patients with aMCI, and 99 age‐and sex‐matched CN controls, as well as a cohort of 142 CN subjects of different ages, were enrolled from Chongqing General Hospital. Plasma CX3CL1 levels were determined using Enzyme‐Linked Immunosorbent Assay (ELISA). Apolipoprotein E genotypes (APOE) were performed. The correlations between Plasma CX3CL1 levels and cognition test scores or age were analyzed. The optimal diagnostic sensitivity and specificity were determined using receiver operating characteristic curve analysis.

**Results:**

Plasma CX3CL1 levels significantly increased with age in CN individuals. No significant sex difference was found. Plasma CX3CL1 levels did not differ significantly between APOE ε4 carriers and non‐carriers. Stepwise elevation across continuum: CX3CL1 levels showed a significant stepwise increase: CN controls (1.73 ± 0.51 ng/mL) < aMCI (2.40 ± 1.06 ng/mL) < AD (4.15 ± 1.24 ng/mL) (*p* < 0.001 between all groups). This pattern persisted in both male and female subgroups, between the AD group and the aMCI group, between the AD group and the CN control group (*p* < 0.001), between the aMCI group and the CN control group, and between the male and female subgroups (*p* < 0.05). CX3CL1 levels negatively correlated with Mini‐Mental State Examination (MMSE) scores and positively correlated with age.

**Conclusions:**

Plasma CX3CL1 levels exhibit a significant age‐dependent increase in cognitively normal individuals, peak in midlife (40–49 years), and demonstrate a stepwise elevation across the AD continuum (CN → aMCI → AD). Strong inverse correlations with cognitive scores in disease groups and high diagnostic accuracy for AD, particularly against CN, support its role as a biomarker reflecting both physiological aging and AD‐related pathological decline. Its regulation appears independent of APOE ε4 status. The midlife peak suggests potential relevance for preclinical processes, warranting further investigation of CX3CL1 as a biomarker and therapeutic target.

**Abbreviations**
AD: Alzheimer's disease
ADL: Activities of Daily Living
aMCI: amnestic Mild Cognitive Impairment
Apo E: Apolipoprotein E genotypes
AUC: Area Under the Curve
Aβ: Amyloid‐beta
Aβ‐PET: Amyloid‐beta Positron Emission Tomography
CDR: Clinical Dementia Rating
CI: Confidence interval
CN: Cognitively Normal
CSF: Cerebrospinal Fluid
CX3CL1: C‐X3‐C motif chemokine ligand 1
CX3CR1: C‐X3‐C motif chemokine receptor 1
ELISA: Enzyme‐Linked Immunosorbent Assay
MMSE: Mini‐Mental State Examination
MoCA: Montreal Cognitive Assessment
PCR: Polymerase Chain Reaction
p‐tau: phosphorylated tau protein
ROC: Receiver Operating Characteristic
SD: Standard Deviation
YKL‐40: Chitinase‐3‐like protein 1

## 1 Introduction

Alzheimer's disease (AD), the leading cause of dementia, poses an escalating global health challenge. Its core neuropathological hallmarks are the accumulation of extracellular amyloid‐beta (Aβ) plaques and intraneuronal neurofibrillary tangles composed of hyperphosphorylated tau protein [1, 2]. While advanced age and carriage of the apolipoprotein E (APOE) ε4 allele are well‐established primary risk factors [3], compelling evidence now implicates chronic neuroinflammation as a critical driver of AD pathogenesis and progression. Within this complex inflammatory milieu, the chemokine CX3CL1 (fractalkine) and its sole receptor, CX3CR1, play multifaceted and often paradoxical roles in AD pathophysiology [4, 5]. CX3CL1‐CX3CR1 signaling is intricately involved in regulating microglial activation states [5, 6], modulating Aβ phagocytosis and clearance, and influencing tau phosphorylation dynamics [7, 8]. These diverse functions position CX3CL1 as a key neuroimmune modulator potentially linking inflammation to core AD pathologies [5, 9, 10].

Despite extensive preclinical investigations, the translational relevance and clinical significance of CX3CL1 signaling in human AD remain poorly defined. Critically, the dynamics of circulating CX3CL1 levels across the entire AD continuum—from cognitively normal aging through amnestic mild cognitive impairment (aMCI) to established AD dementia—and their relationship to age‐related cognitive trajectories have not been systematically evaluated. Furthermore, existing clinical studies report conflicting findings regarding alterations of plasma or serum CX3CL1 in AD patients, with reports of increases, decreases, or no change. These inconsistencies likely stem from methodological variations (e.g., sample handling, assay differences), cohort heterogeneity (e.g., diagnostic criteria, comorbidities), and crucially, inadequate control for powerful confounding factors like age and sex.

To bridge these critical knowledge gaps, we conducted a comprehensive analysis of plasma CX3CL1 levels within a large, well‐characterized Chinese cohort spanning the cognitive spectrum. Specifically, this study aimed to: (i) Establish baseline dynamics: Characterize age‐ and sex‐associated variations in plasma CX3CL1 levels among cognitively normal (CN) individuals across a broad adult age range. (ii) Determine diagnostic utility: Investigate the pattern of plasma CX3CL1 alterations through sex‐stratified comparisons across the AD continuum (CN controls vs. aMCI vs. AD). (iii) Evaluate clinical relevance: Assess the correlations between plasma CX3CL1 levels and established clinical markers, including cognitive function (Mini‐Mental State Examination, MMSE), global disease severity (Clinical Dementia Rating, CDR), functional status (Activities of Daily Living, ADL), and education level. (iv) Assess biomarker performance: Quantify the diagnostic accuracy of plasma CX3CL1 for distinguishing AD, aMCI, and CN states using receiver operating characteristic (ROC) curve analysis.

This study provides novel insights into plasma CX3CL1 as a neuroinflammation‐associated biomarker reflecting both physiological aging processes and pathological cognitive decline along the AD continuum.

## 2 Methods

A total of 443 participants were enrolled in this study, including 142 cognitively normal (CN) individuals across a wider age range, and 130 patients with Alzheimer's disease (AD), 72 patients with amnestic mild cognitive impairment (aMCI), and 99 CN controls. All participants were consecutively enrolled between January 2019 and March 2025. The study protocol was approved by the Institutional Review Board of Chongqing General Hospital, and written informed consent was obtained from all participants or their legal representatives.

### 2.1 Clinical Assessment

A total of 443 participants, including 142 cognitively normal (CN) individuals across a wider age range, and 130 patients with Alzheimer's disease (AD), 72 patients with amnestic mild cognitive impairment (aMCI), and 99 CN controls were recruited from the Department of Geriatrics, Chongqing General Hospital (Chongqing Geriatrics Clinical Research Center). Another group of CN subjects was recruited from the Chongqing General Hospital Physical Examination Center. Exclusion criteria are as follows: (i) family history of dementia; (ii) substance abuse; (iii) history of major psychiatric illness that may impair cognitive ability, such as persistent psychosis, major depressive disorder, bipolar disorder, or schizophrenia; (iv) history of traumatic brain injury or other comorbid neurologic disorders; and (v) major medical illness such as severe heart, lung, liver, or kidney disease or any type of tumors.

All participants underwent a standardized clinical assessment including medical history, physical examination, neuropsychological assessment and laboratory tests. Participants' cognitive and functional status was assessed using a neuropsychological suite of instruments, including the Chinese version of the Brief Mental State Examination (MMSE), the Montreal Cognitive Assessment (MoCA Beijing version), and the Activity of Daily Living Scale (ADL). Following the widely adopted education‐adjusted norms in China, the cut‐offs for defining abnormal cognitive screening results in our study are: MMSE: ≤ 17 points for illiterate individuals; ≤ 22 points for those with primary education; ≤ 24 points for those with secondary education or higher. MoCA (Beijing version): ≤ 13 points for illiterate individuals; ≤ 19 points for those with primary education; ≤ 24 points for those with secondary education or higher. Individuals with abnormal results on the MMSE or MoCA underwent a series of additional neuropsychological evaluations, including the Auditory Verbal Learning Test, the Drawing Clock Test, the Connectivity Test, the Boston Test, the Digit Breadth Test, the Clinical Dementia Rating (CDR) Scale, the Pfeiffer Outpatient Disability Questionnaire, and the Hachinski Ischemia Score. Subjects with abnormal cognitive function underwent brain computed tomography/magnetic resonance imaging scans and blood tests for thyroxine, vitamin B12, folic acid, and human immunodeficiency virus/syphilis to rule out metabolic and infectious causes of cognitive decline.

The clinical diagnosis of aMCI was made according to the Petersen (Mayo Clinic) criteria (Petersen RC, 2004), requiring: (1) memory complaint (preferably corroborated), (2) objective memory impairment for age and education, (3) largely preserved general cognitive function, (4) essentially intact activities of daily living, and (5) not meeting dementia criteria [11]. Dementia was diagnosed based on criteria modified from the Diagnostic and Statistical Manual of Mental Disorders, Fourth Edition. AD was diagnosed according to the criteria of the National Institute of Neurological and Communicative Diseases and Stroke/AD and Related Disorders Association [12]. Non‐amnestic MCI individuals were excluded to maintain a homogeneous cohort focused on the amnestic phenotype most specific to the AD continuum [12].

### 2.2 Plasma Sample Preparation

For all participants, fasting blood collection was scheduled on the same day as the cognitive assessment. This protocol was strictly followed in the vast majority of cases; in the rare instances where same‐day collection was logistically unfeasible, blood was drawn the following morning after an overnight fast, ensuring a maximum interval of 24 h. No systematic difference in this sampling interval was observed across diagnostic groups. Fasting venous blood samples were collected into 5 mL Ethylenediaminetetraacetic Acid (EDTA) tubes and centrifuged at 3000 rpm for 10 min at 4°C. Plasma was separated by removing non‐plasma components, aliquoted into Eppendorf tubes (EP) tubes, and stored at −80°C until analysis. Routine blood tests—including liver function, renal function, thyroid function, vitamin B12, folate, and screening for human immunodeficiency virus (HIV) and syphilis—were performed in our laboratory.

Plasma concentrations of CX3CL1 were measured using a commercially available enzyme‐linked immunosorbent assay (ELISA) kit (R&D Systems, Minneapolis, Minnesota, USA). APOE genotyping (E2/E3/E4 alleles) was conducted using a fluorescence‐based polymerase chain reaction (PCR) method with a commercially available kit (Xiamen Rendu Biotech Co. Ltd., China).

### 2.3 Statistical Analysis

Statistical analysis was performed using SPSS 27.0 software. Normality of data distribution was assessed using the Kolmogorov–Smirnov test. For comparisons of continuous variables across two or more groups, one‐way analysis of variance (ANOVA) was used for normally distributed data, and the Kruskal‐Wallis test was used for non‐normally distributed data. When adjustment for covariates (e.g., age) was required, analysis of covariance (ANCOVA) was applied to normally distributed data, whereas a rank‐based nonparametric ANCOVA was applied to non‐normally distributed data. Measurements are expressed as mean ± standard deviation (^−^x ± s). Correlations between plasma CX3CL1 levels and cognitive scale scores or age were analyzed using Pearson and Spearman's correlation analysis. Differences between the two groups were compared using chi‐square tests. Predictive value was assessed using subject work characteristic (ROC) curves. Logistic regression analysis was used for multifactor tests.

### 2.4 Availability of Data and Materials

Anonymized data will be shared at the request of qualified researchers.

## 3 Results

### 3.1 Plasma CX3CL1 Levels in CN Subjects

**FIGURE 1:**
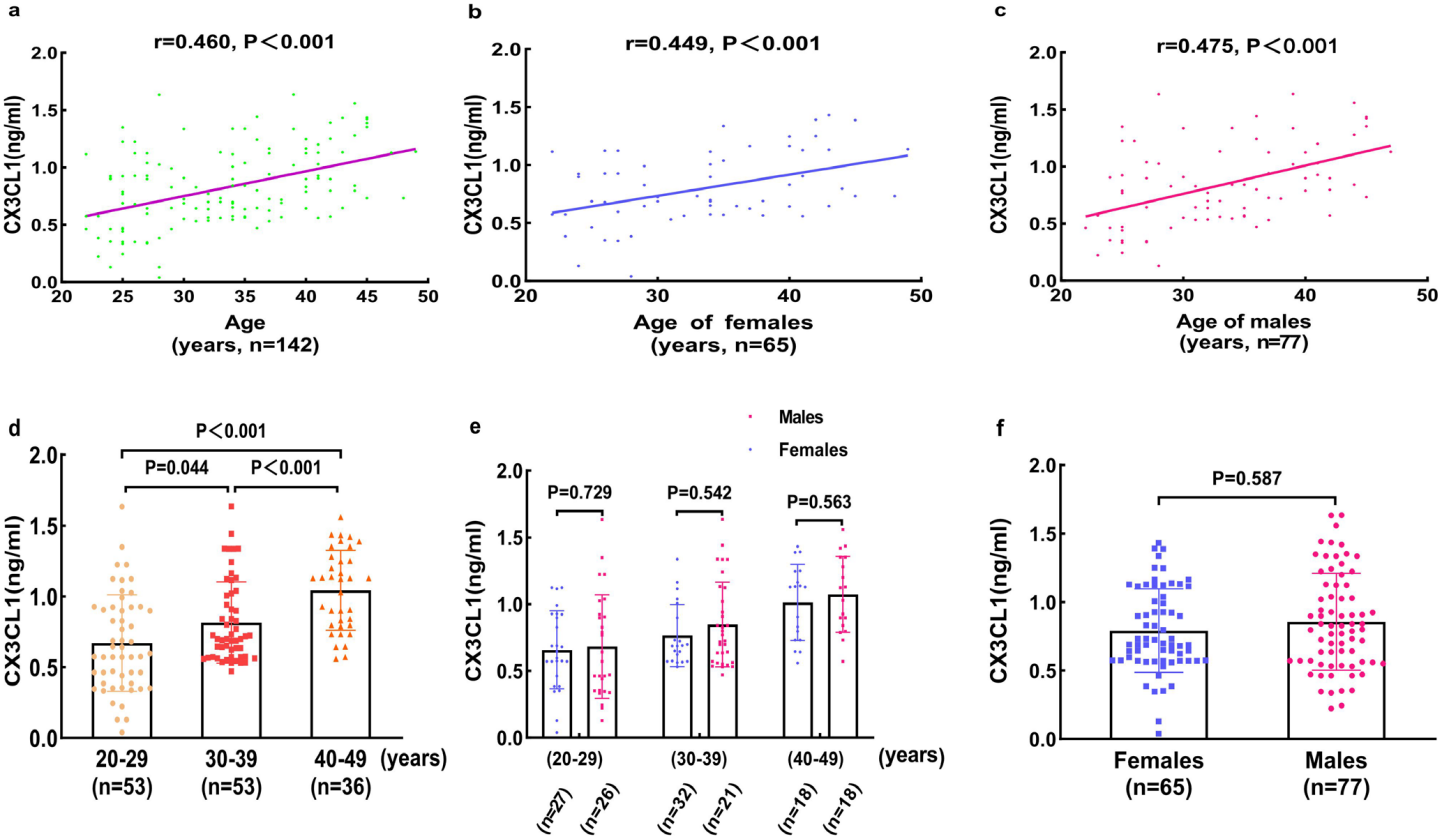
Plasma CX3CL1 levels in a cognitively normal (CN) cohort, further divided into specific age and gender categories. (a) Analysis of the correlation between plasma CX3CL1 levels and age across all subjects in the CN cohort. (b) Analysis of the correlation between plasma CX3CL1 levels and age in female CN participants. (c) Analysis of the correlation between plasma CX3CL1 levels and age in male CN participants. (d) Comparison of plasma CX3CL1 levels in CN participants according to different age groups. (e) Comparison of plasma CX3CL1 levels in male and female CN participants in different age groups. (f) Comparison of plasma CX3CL1 levels in female and male CN participants.

This study aimed to examine the correlation between age and plasma CX3CL1 levels. A total of 142 cognitively normal (CN) individuals across a wide age spectrum were recruited (Table 1). The findings revealed an increase in plasma CX3CL1 levels with age, demonstrating a significant positive correlation (*r* = 0.460, *p* < 0.001; Figure 1a). This correlation was also notably present in both male (*r* = 0.475, *p* < 0.001; Figure 1c) and female (*r* = 0.449, *p* < 0.001; Figure 1b) sub‐groups, respectively.

**TABLE 1:** Age and sex of the cognitively normal subjects.

Age range (years), *N* = 142	Average age (years)			Sex		
	Females, *n* = 65	Males, *n* = 77	*p*	Females, *n* = 65 (45.8%)	Males, *n* = 77 (54.2%)	*p*
20–29, *n* = 53	25.42 ± 2.21	25.67 ± 1.84	0.659	26 (49.1%)	27 (50.9%)	0.523
30–39, *n* = 53	34.43 ± 2.40	34.34 ± 2.77	0.909	21 (39.6%)	32 (60.4%)	
40–49, *n* = 36	42.67 ± 2.68	42.94 ± 2.18	0.521	18 (50.0%)	18 (50.0%)	

Participants were grouped according to age, with the lowest plasma CX3CL1 levels observed in the 20–29 years age group, increasing to a peak in the 40–49 years age group. There was no statistically significant difference between the 20–29 and 30–39 years age groups. However, the plasma CX3CL1 levels in the 40–49 years age group were significantly higher than those in both the 20–29 and 30–39 years age groups (Figure 1d).

In terms of gender differences, no significant variation was observed in the mean age between female (*n* = 65) and male (*n* = 77) participants, with ages of 33.11 ± 7.48 years and 33.31 ± 6.97 years respectively (*p* = 0.795). Similarly, the plasma CX3CL1 levels showed no significant difference between females and males, with respective levels of 0.79 ± 0.31 ng/mL and 0.84 ± 0.36 ng/mL (*p* = 0.587) (Figure 1f).

We determined the APOE genotype and analyzed plasma CX3CL1 levels in both carriers and non‐carriers of the APOE ε4 allele. Our findings revealed no significant differences in plasma CX3CL1 levels between ε4 carriers and non‐carriers (data not shown).

### 3.2 Plasma CX3CL1 Levels in AD and aMCI Patients and CN Subjects

To examine plasma CX3CL1 variations, we enrolled 130 Alzheimer's disease (AD), 72 amnestic mild cognitive impairment (aMCI), and 99 cognitively normal (CN) participants. Demographics and clinical profiles are in Table 2. As expected, the AD group had the lowest Mini‐Mental State Examination (MMSE) and Activity of Daily Living (ADL) scores, and the highest Clinical Dementia Rating (CDR) scores.

**TABLE 2:** Demographic and clinical data of the patients with Alzheimer's disease (AD) and amnestic mild cognitive impairment (aMCI), and cognitively normal (CN) subjects.

Clinical variable	AD (*n* = 130)	aMCI (*n* = 72)	CN (*n* = 99)	*p*
Age (years)	86.51 ± 10.30	86.24 ± 10.28	76.83 ± 12.33	< 0.001
50–87 (years)	76.89 ± 9.43 (*n* = 53)	77.13 ± 9.17 (*n* = 31)	72.69 ± 9.83 (*n* = 80)	0.013
88–105 (years)	93.13 ± 3.20 (*n* = 77)	93.12 ± 3.41 (*n* = 41)	94.26 ± 2.78 (*n* = 19)	0.273
Female, *n* (%)	60 (46.2%)	33 (45.8%)	47 (47.5%)	0.972
	85.90 ± 10.87	88.12 ± 9.28	76.40 ± 13.39	< 0.001
Male, *n* (%)	70 (53.8%)	39 (54.2%)	52 (52.5%)	0.972
	87.03 ± 9.84	84.64 ± 10.92	77.21 ± 11.42	< 0.001
Education, (years)	9.79 ± 4.11	9.88 ± 4.34	10.19 ± 3.94	0.736
MMSE score	10.58 ± 9.22	16.56 ± 7.18	25.91 ± 2.52	< 0.001
CDR score	2.22 ± 0.88	1.50 ± 0.81	0 ± 0.58	< 0.001
ADL score	32.12 ± 34.77	46.04 ± 36.62	80.76 ± 28.76	< 0.001
Hypertension, *n* (%)	106 (81.5%)	54 (75.0%)	65 (65.7%)	0.023
Diabetes mellitus, *n* (%)	57 (43.8%)	34 (47.2%)	45 (45.5%)	0.897
Coronary atherosclerotic heart disease, *n* (%)	66 (50.8%)	48 (66.7%)	36 (36.4%)	< 0.001
Chronic obstructive pulmonary disease, *n* (%)	23 (17.7%)	21 (29.2%)	17 (17.2%)	0.098

Abbreviations: AD, Alzheimer's disease; ADL, Activities of Daily Living; aMCI, amnestic mild cognitive impairment; CDR, Clinical Dementia Rating; CN, cognitively normal controls; MMSE, Mini‐Mental State Examination; SD, standard deviation.

**FIGURE 2:**
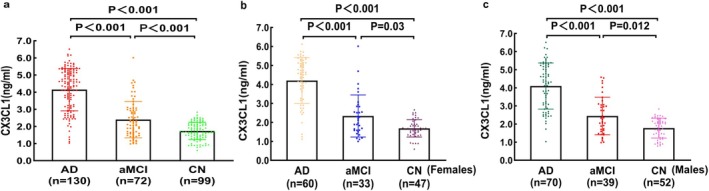
Plasma CX3CL1 levels in subjects with Alzheimer's disease (AD), amnestic mild cognitive impairment (aMCI), and cognitively normal (CN) controls. (a) Comparison of plasma CX3CL1 levels in AD, aMCI, and CN groups. (b) Comparison of plasma CX3CL1 levels in female participants of the AD, aMCI, and CN groups. (c) Comparison of plasma CX3CL1 levels in male participants of the AD, aMCI, and CN groups.

Age‐adjusted analysis revealed a significant stepwise increase in plasma CX3CL1 levels across the cognitive spectrum from cognitively normal (CN) to amnestic mild cognitive impairment (aMCI) and Alzheimer's disease (AD) groups (*F* = 137.50, *p* < 0.001). Post hoc comparisons showed that the AD group had significantly higher levels (4.15 ± 1.24 ng/mL) than both the aMCI (2.40 ± 1.06 ng/mL, *p* < 0.001) and CN (1.73 ± 0.51 ng/mL, *p* < 0.001) groups, and that the aMCI group levels were also significantly higher than those of the CN group (*p* < 0.001) (Figure 2a).

This diagnostic pattern remained robust after stratification by sex. Among females, age‐adjusted analysis indicated a significant main effect of diagnosis (*F* = 67.24, *p* < 0.001), with levels in AD patients (4.21 ± 1.20 ng/mL) being significantly higher than in those with aMCI (2.34 ± 1.11 ng/mL, *p* < 0.001) and CN participants (1.47 ± 0.55 ng/mL, *p* < 0.001) (Figure 2b). Similarly, a significant age‐adjusted main effect was observed in males (*F* = 67.07, *p* < 0.001), with higher levels in the AD group (4.10 ± 1.27 ng/mL) compared to both the aMCI (2.44 ± 1.04 ng/mL, *p* < 0.001) and CN (1.77 ± 0.55 ng/mL, *p* < 0.001) groups (Figure 2c).

**FIGURE 3:**
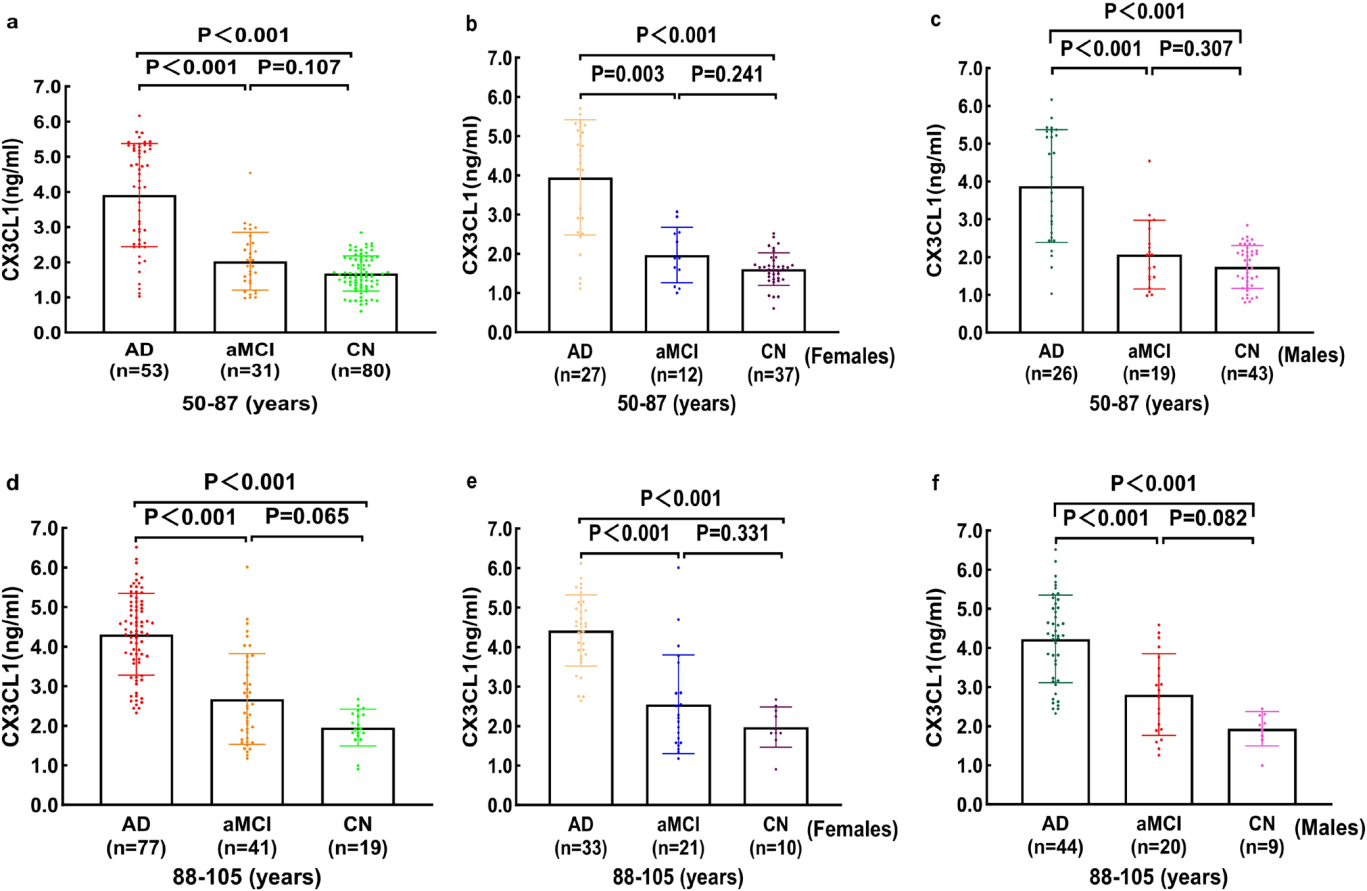
Plasma CX3CL1 levels in Alzheimer's disease (AD) patients, amnestic mild cognitive impairment (aMCI) patients, and cognitively normal (CN) controls. (a) Comparison of plasma CX3CL1 levels in AD, aMCI, and CN participants aged 50–87 years. (b) Comparison of plasma CX3CL1 levels in female participants in the three diagnostic groups aged 50–87 years. (c) Comparison of plasma CX3CL1 levels in male participants in the three diagnostic groups aged 50–87 years. (d) Comparison of plasma CX3CL1 levels in participants with AD, aMCI, and CN aged 88–105 years. (e) Comparison of plasma CX3CL1 levels in female participants in the three diagnostic groups aged 88–105 years. (f) Comparison of plasma CX3CL1 levels in male participants in the three diagnostic groups aged 88–105 years.

We further analyzed the subset of participants aged 50–87 years. Age‐adjusted plasma CX3CL1 levels differed significantly across diagnostic groups (*F* = 46.55, *p* < 0.001). Levels were significantly higher in AD patients (3.91 ± 1.47 ng/mL) than in both aMCI (2.03 ± 0.83 ng/mL, *p* < 0.001) and CN (1.68 ± 0.50 ng/mL, *p* < 0.001) participants (Figure 3a). This pattern was consistently observed in both sexes: among females (age‐adjusted main effect: *F* = 23.42, *p* < 0.001) and males (age‐adjusted main effect: *F* = 20.97, *p* < 0.001). Notably, within this age bracket, post hoc comparisons revealed no significant difference in CX3CL1 levels between the aMCI and CN groups for either sex (Figure 3b,c).

Similarly, in the 88–105 age group, CX3CL1 levels were significantly higher in AD vs. aMCI (4.31 ± 1.03 ng/mL vs. 2.67 ± 1.15 ng/mL, *p* < 0.001) and CN (4.31 ± 1.03 ng/mL vs. 1.96 ± 0.46 ng/mL, *p* < 0.001) (Figure 3d). Sex stratification confirmed this: levels were significantly higher in AD females vs. aMCI females (4.42 ± 0.90 ng/mL vs. 2.55 ± 1.25 ng/mL, *p* < 0.001) and CN females (4.42 ± 0.90 ng/mL vs. 1.97 ± 0.51 ng/mL, *p* < 0.001) (Figure 3e), and in AD males vs. aMCI males (4.23 ± 1.12 ng/mL vs. 2.81 ± 1.04 ng/mL, *p* < 0.001) and CN males (4.23 ± 1.12 ng/mL vs. 1.94 ± 0.44 ng/mL, *p* < 0.001) (Figure 3f). Consistent with the 50–87 age range, no significant difference was found between aMCI and CN groups within either sex (Figure 3e,f).

### 3.3 Associations Between Plasma CX3CL1 Levels and Cognitive Assessment Scores

In order to elucidate the association between plasma CX3CL1 concentrations and cognitive performance, a comprehensive analysis was conducted across our entire cohort. Additionally, we performed a nuanced stratification of this analysis based on disease status. This included distinct groups characterized by Alzheimer's disease (AD), amnestic mild cognitive impairment (aMCI), and cognitively normal (CN) individuals.

**FIGURE 4:**
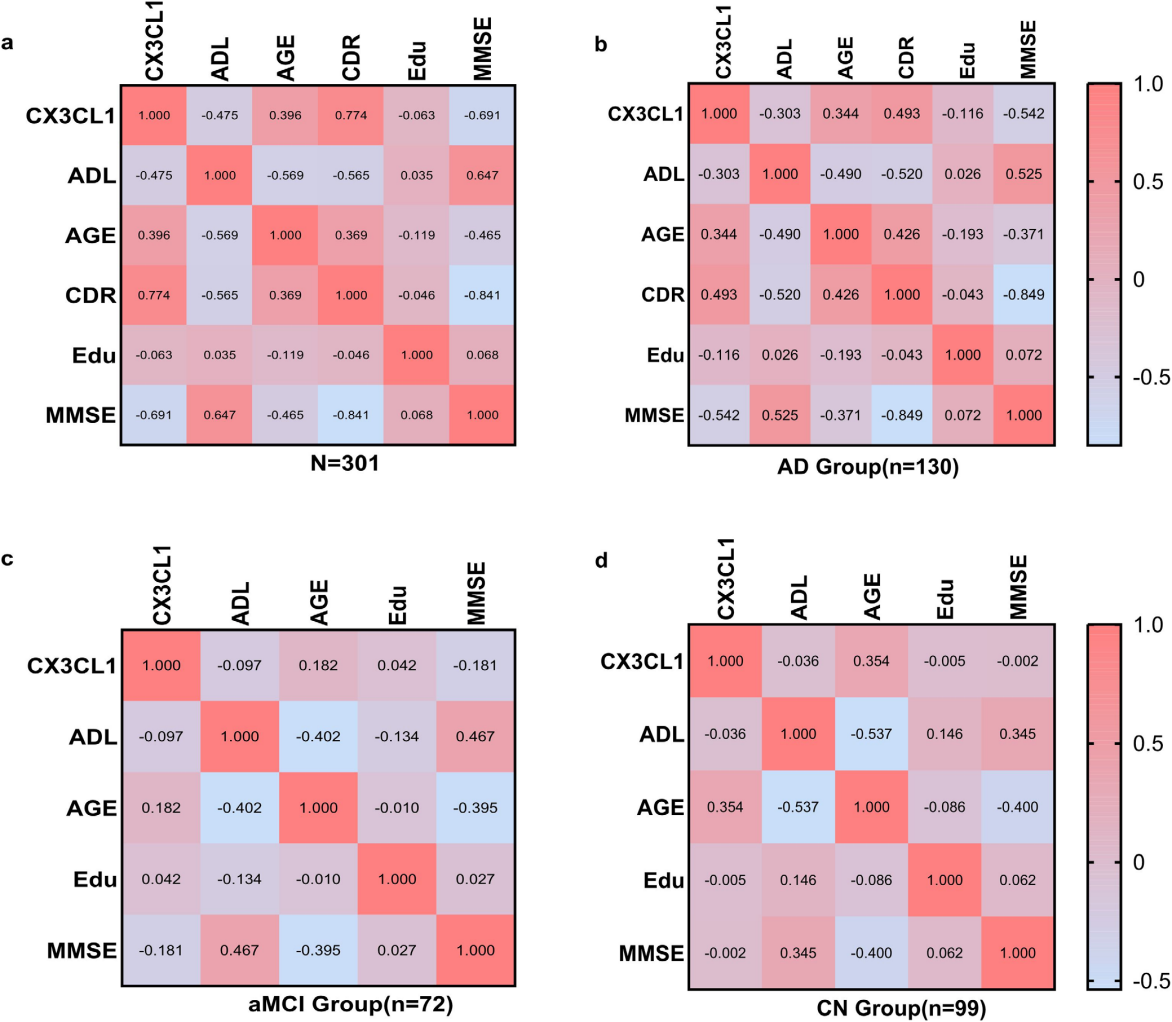
The correlation matrix shows the Spearman correlation between plasma CX3CL1 levels and ADL scores, age, CDR scores, educational level, and MMSE scores in the cohort, including the AD, aMCI, and CN groups. All groups (a), AD group (b), aMCI group (c), and CN group (d).

**FIGURE 5:**
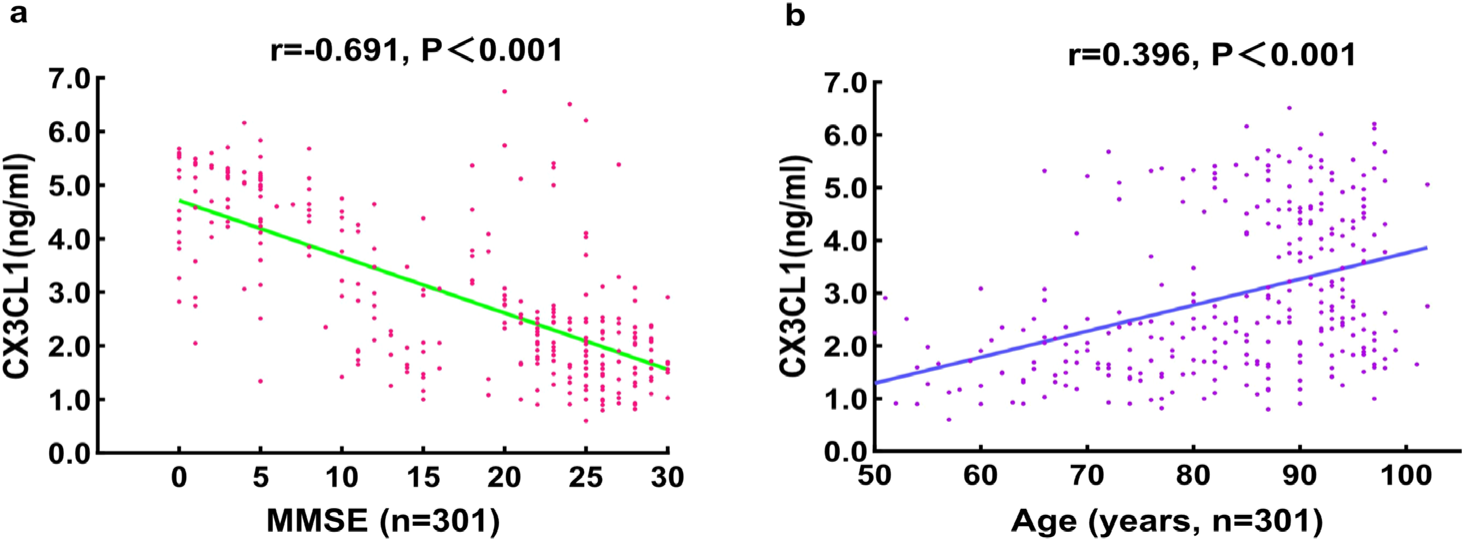
Correlation between plasma CX3CL1 levels in all cohorts and MMSE scores and age. MMSE scores (a) and age (b).

In the total cohort, plasma CX3CL1 levels had complicated relationships with cognitive assessment indicators and demographic variables (Figures 4a and 5). Plasma CX3CL1 levels were negatively associated with the MMSE scores (*r* = −0.691, *p* < 0.001), which meant that higher CX3CL1 levels were accompanied by lower cognitive performance (Figures 4a and 5a). Positive correlations were observed between CX3CL1 levels and age (*r* = 0.396, *p* < 0.001), indicating that older participants were inclined to have higher CX3CL1 levels (Figures 4a and 5b).

In the AD group, plasma CX3CL1 levels were inversely associated with MMSE scores (*r* = −0.542, *p* < 0.001), indicating increased cognitive impairment with higher CX3CL1 concentrations. Furthermore, there was a positive correlation between CX3CL1 levels and the Clinical Dementia Rating (CDR; *r* = 0.493, *p* < 0.001), suggesting a more severe disease state with elevated CX3CL1 concentrations (Figure 4b). In the aMCI group, plasma CX3CL1 levels displayed a negative correlation with MMSE scores (*r* = −0.181, *p* = 0.128), consistent with diminished cognitive performance at elevated CX3CL1 concentrations (Figure 4c). Among the cognitively normal (CN) participants, there were no significant relationships between CX3CL1 levels and MMSE scores or other cognitive/demographic variables (Figure 4d). This implies that CX3CL1 might have disease‐specific impacts in pathological cohorts (AD, aMCI).

### 3.4 ROC Curve Analysis of the Diagnostic Value of Plasma CX3CL1 Levels for AD and aMCI

**FIGURE 6:**
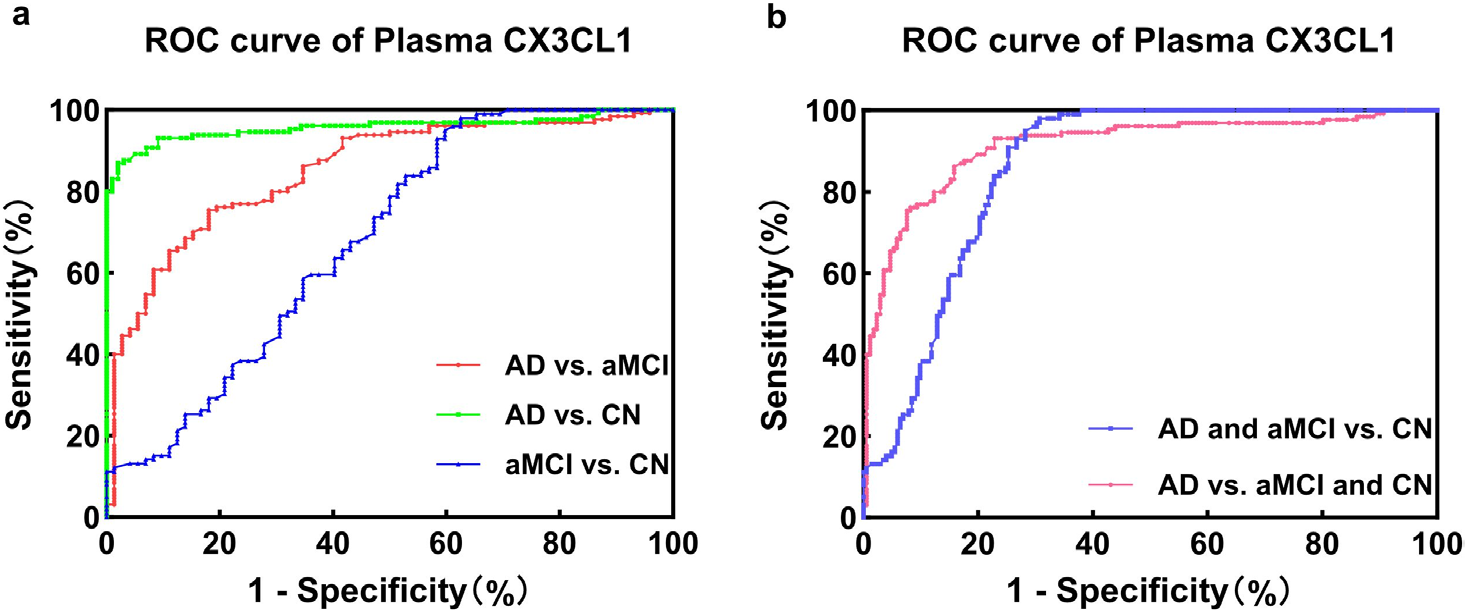
Receiver operating characteristic (ROC) curve for plasma CX3CL1. (a) The ROC curve for plasma CX3CL1 in discriminating AD from aMCI, AD from CN, and aMCI from CN. (b) The ROC curve for plasma CX3CL1 in discriminating AD and aMCI from CN, AD from aMCI, and CN.

The area under the receiver operating characteristic curve (AUC) for plasma CX3CL1 in distinguishing AD from CN was calculated at 0.958 (95% confidence intervals [CI], 0.930–0.985). With the CN toff value optimized via Youden's index, plasma CX3CL1 achieved 86.9% sensitivity and 98% specificity in identifying AD versus CN (refer to Figure 6a and Table 3 for details). The diagnostic accuracy of plasma CX3CL1 for distinguishing AD from aMCI was indicated by an AUC of 0.852, with sensitivities and specificities reaching 75.4% and 81.9%, respectively (Figure 6a and Table 3). The AUC for differentiating aMCI from CN subjects was 0.681, with sensitivity and specificities of 37.5% and 98%, respectively (Figure 6a and Table 3).

**TABLE 3:** Discriminative values of plasma CX3CL1 using an ROC curve analysis.

ROC analysis	AD vs. CN	AD vs. aMCI	aMCI vs. CN	CN vs. AD & aMCI	AD vs. aMCI & CN
AUC	0.958	0.852	0.681	0.859	0.913
*p*	< 0.001	< 0.001	< 0.001	< 0.001	< 0.001
95% CI	0.930–0.985	0.797–0.906	0.597–0.766	0.819–0.900	0.878–0.948
Cutoff, ng/mL	2.54	3.122	2.54	2.54	2.57
Sensitivity, %	86.9	75.4	37.5	69.3	86.2
Specificity, %	98	81.9	98	98	84.2

Abbreviations: AUC, Area Under the Curve; CI, Confidence interval; CX3CL1, Inflammation chemokine; ROC, Receiver Operating Characters.

We conducted additional analyzes to assess the capacity of plasma CX3CL1 to distinguish AD from both aMCI and CN subjects (Figure 6b and Table 3). The AUC for this comparison was 0.913 (95% CI, 0.878–0.948), with sensitivities and specificities of 86.2% and 84.2%, respectively. Similarly, we evaluated the potential of CX3CL1 to differentiate AD and aMCI from the CN groups (Figure 6b and Table 3). The resultant AUC was 0.859 (95% CI, 0.819–0.900), accompanied by sensitivities and specificities of 69.3% and 98%, respectively.

## 4 Discussion

To our knowledge, this study provides the first comprehensive analysis of plasma CX3CL1 dynamics across both physiological aging and the AD continuum (CN → aMCI → AD) within a Chinese cohort. Our key findings demonstrate: (i) a significant age‐dependent increase in plasma CX3CL1 levels among cognitively normal (CN) individuals; (ii) a stepwise elevation in CX3CL1 concentrations across the AD diagnostic spectrum (CN: 1.73 ± 0.51 ng/mL; aMCI: 2.40 ± 1.06 ng/mL; AD: 4.15 ± 1.24 ng/mL; *p* < 0.001 between all groups); and (iii) robust inverse correlations between plasma CX3CL1 levels and cognitive performance (MMSE scores) specifically within the aMCI and AD groups.

In our preceding study, we observed an age‐related increase in urinary CX3CL1 [13], which aligned with the trends noted in plasma CX3CL1. Notably, this elevation was also evident in patients with AD, further underscoring the consistency between urinary and plasma CX3CL1 levels. The observed age‐related increase in plasma CX3CL1 in CN subjects aligns with its recognized role in inflammaging [14, 15], providing further mechanistic support for aging as a primary AD risk factor [16, 17]. Critically, the peak concentration observed in the 40–49 years age group suggests CX3CL1 may be implicated in preclinical pathological processes preceding overt cognitive symptoms [18, 19, 20]. This midlife elevation warrants further investigation as a potential biomarker for early AD risk stratification or a target for preventive interventions [21].

Our findings across the AD continuum corroborate the majority of clinical studies reporting elevated CSF or peripheral blood CX3CL1 levels in AD and MCI compared to CN controls [22], although discrepancies exist in the literature reporting decreased or unchanged levels. The robust, stepwise increase observed here may be attributed to methodological strengths, including ethnic homogeneity within our cohort, rigorous exclusion of individuals with inflammatory comorbidities, and standardized plasma processing protocols, minimizing confounding variables that have likely contributed to prior inconsistencies.

Notably, plasma CX3CL1 levels showed no significant association with APOE ε4 carrier status, despite ε4's well‐established role as the strongest genetic risk factor for AD [23, 24]. This dissociation suggests CX3CL1 dysregulation occurs predominantly through pathways independent of the APOE ε4 allele [25]. Mechanistically, this points towards regulation driven by age‐associated neuroinflammation or tau pathology‐mediated mechanisms [26], rather than being secondary to Aβ‐centric pathways strongly linked to APOE ε4. This APOE‐independent regulation underscores CX3CL1's potential as a complementary biomarker, reflecting distinct neuroinflammatory cascades central to AD pathogenesis, particularly those involving tau phosphorylation and microglial activation [27, 28].

The observed age‐dependent rise in plasma CX3CL1 likely reflects a compensatory, homeostatic response within the aging brain. Under physiological conditions, neuron‐derived CX3CL1, via engagement with its microglial receptor CX3CR1, serves as a critical “off” signal, dampening excessive microglial activation and thereby exerting net anti‐inflammatory and neuroprotective effects—a process integral to “inflammaging” [15, 29]. However, within the AD pathological continuum, this homeostatic balance is profoundly disrupted. Amyloid‐beta (Aβ) plaques and tau tangles drive a sustained activation of microglia and astrocytes, which in turn become major sources of CX3CL1 [30, 31]. In this context, chronic elevation of CX3CL1, particularly its soluble form (sCX3CL1), may contribute to a maladaptive neuroinflammatory loop. While it may initially facilitate Aβ phagocytosis, persistent CX3CL1‐CX3CR1 signaling has been implicated in exacerbating tau hyperphosphorylation, impairing tau clearance, and amplifying pro‐inflammatory cytokine release [10, 32]. Thus, the stepwise increase in plasma CX3CL1 from CN to aMCI to AD may mark the transition of this signaling axis from a protective homeostatic mechanism to a contributor to synergistic gliosis and neuronal damage.

When considering the landscape of inflammatory biomarkers in AD, CX3CL1 presents distinct advantages over classical systemic cytokines such as IL‐6, IL‐1β, and TNF‐α. While these interleukins are valuable indicators of generalized inflammation, their peripheral levels can be confounded by non‐CNS comorbidities [33]. CX3CL1, in contrast, is more neuron‐specific. Its primary neuronal origin and exclusive signaling through microglial CX3CR1 position it as a direct proxy for the neuron‐microglial communication axis in AD neuroinflammation [16, 33]. Furthermore, its involvement in both Aβ clearance and tau pathology modulation ties it more closely to core AD pathogenic cascades than cytokines with broader roles. Compared to other glial markers like Chitinase‐3‐like protein 1 (YKL‐40), which shows elevation in AD, CX3CL1's stepwise dynamic increase across the CN‐aMCI‐AD continuum (as evidenced here) may offer finer discriminatory power for disease staging [20]. Although large‐scale validation is still needed and its therapeutic modulation requires caution due to its dual roles, CX3CL1 emerges as a promising pathology‐proximal biomarker that complements rather than replaces existing inflammatory panels, potentially enhancing early detection and tracking of disease progression.

Several limitations of this study should be acknowledged. First, clinical diagnoses relied on established criteria but lacked confirmation with gold‐standard amyloid (Aβ‐PET or CSF Aβ42) or tau (tau‐PET or CSF p‐tau) biomarkers. While previous studies suggest correlations between plasma and CSF CX3CL1 [27, 28], simultaneous CSF‐plasma measurements in our cohort would have strengthened the interpretation of peripheral CX3CL1 levels in relation to central nervous system pathology. Second, the cross‐sectional design precludes causal inference regarding CX3CL1's role in disease progression. Third, investigating correlations with neuroinflammatory imaging (e.g., microglial activation via TSPO‐PET) and other key inflammatory mediators would provide deeper mechanistic insights [34, 35]. Fourth, we did not systematically collect data on medication use, particularly anti‐inflammatory agents (e.g., NSAIDs), which could potentially influence plasma CX3CL1 levels [33, 36]. Future prospective studies should carefully document and adjust for such factors to confirm the robustness of our findings. Finally, it remains unclear whether modulating CX3CL1/CX3CR1 signaling could alter disease trajectory [37, 38].

Future research directions are critical. Validation of plasma CX3CL1 against established AD biomarkers (tau‐PET, Aβ‐PET, CSF p‐tau/Aβ42) is essential to define its relationship to core pathologies. Longitudinal studies are urgently needed to determine whether elevated CX3CL1 levels in midlife predict subsequent conversion to aMCI or AD. Furthermore, exploring the therapeutic potential of modulating the CX3CL1/CX3CR1 axis, perhaps targeting the observed midlife peak, represents a promising avenue for preventive strategies.

## 5 Conclusion

In summary, our findings establish plasma CX3CL1 as a robust biomarker reflecting both physiological aging processes and pathological progression along the AD continuum within a Chinese population. Its significant age‐dependent increase peaking in midlife, stepwise elevation from CN through aMCI to AD, strong correlation with cognitive decline in disease states, high diagnostic accuracy, and regulation independent of the major AD genetic risk factor APOE ε4 collectively support its potential clinical utility for screening and monitoring disease progression. The midlife peak in CX3CL1 levels highlights a critical window warranting investigation for targeted preventive interventions.

## Author Contributions

**Ling Wang:** conceptualization, data curation, methodology, resources, writing – original draft, writing – review and editing. **Yujie Liu:** data curation, methodology, writing – review and editing. **Fei Li:** data curation, formal analysis, project administration, resources, writing – review and editing. **Xuelin Li:** data curation, investigation, resources, writing – review and editing. **Lanlan Li:** data curation, methodology, resources, writing – review and editing. **Jie Zhang:** conceptualization, data curation, writing – review and editing. **Yali Xu:** conceptualization, data curation, funding acquisition, resources, writing – original draft, writing – review and editing.

## Funding

This work was supported by the Natural Science Foundation of Chongqing (Grant CSTB2024NSCQ‐MSX0945 to YX).

## Conflicts of Interest

The authors declare no conflicts of interest.

## Data Availability

The data that support the findings of this study are available from the corresponding author upon reasonable request.
